# Baseline Obesity Increases 25-Year Risk of Mortality due to Cerebrovascular Disease: Role of Race

**DOI:** 10.3390/ijerph16193705

**Published:** 2019-10-01

**Authors:** Shervin Assari, Mohsen Bazargan

**Affiliations:** 1 Department of Family Medicine, Charles R. Drew University of Medicine and Science, Los Angeles, CA 90059, USA; Mohsenbazargan@cdrewu.edu; 2 Department of Family Medicine, University of California, Los Angeles (UCLA), Los Angeles, CA 90095, USA

**Keywords:** Stroke, body mass index, race, ethnicity, mortality

## Abstract

*Background*: Although obesity may have a role as a risk factor for cerebrovascular mortality, less is known about how demographic and social groups differ in this regard. *Aims*: This study had two aims: first to investigate the predictive role of baseline obesity on long-term risk of mortality due to cerebrovascular disease, and second, to test racial variation in this effect. *Methods*: the Americans’ Changing Lives Study (ACL) 1986–2011 is a state of the art 25-year longitudinal cohort study. ACL followed a nationally representative sample of Blacks (*n* = 1156) and Whites (*n* = 2205) for up to 25 years. Baseline obesity was the main predictor of interest, time to cerebrovascular death was the main outcome of interest. Demographic characteristics, socioeconomic status (educational attainment and household income), health behaviors (exercise and smoking), and health (hypertension and depressive symptoms) at baseline were covariates. Cox proportional hazards models were used to test additive and multiplicative effects of obesity and race on the outcome. *Results*: From the total 3,361 individuals, 177 people died due to cerebrovascular causes (Whites and Blacks). In the pooled sample, baseline obesity did not predict cerebrovascular mortality (hazard ratio (HR) = 0.86, 0.49–1.51), independent of demographic, socioeconomic, health behaviors, and health factors at baseline. Race also interacted with baseline obesity on outcome (HR = 3.17, 1.09–9.21), suggesting a stronger predictive role of baseline obesity on cerebrovascular deaths for Black people compared to White individuals. According to the models that were run specific to each race, obesity predicted risk of cerebrovascular mortality for Blacks (HR = 2.51, 1.43–4.39) but not Whites (HR = 0.69, 0.31–1.53). *Conclusions*: Baseline obesity better predicts long-term risk of cerebrovascular death in Black individuals compared to White people. More research should explore factors that explain why racial differences exist in the effects of obesity on cerebrovascular outcome. Findings also have implications for personalized medicine.

## 1. Introduction

### 1.1. Background

Cerebrovascular conditions such as stroke are leading contributing factors to both morbidity and also mortality in the United States (U.S.) and globally [1,2]. Stroke is the second most common cause of death in the world [3,4]. Stroke kills 5.5 million people and causes 44 million disability-adjusted life-years (DALYs) lost annually [3,4,5]. Stroke is associated with considerable societal and health care costs due to short-term (in-hospital care, critical care) and long-term care, as well as indirect costs [6]. Epidemiological studies are needed to gain the knowledge that is required for reducing the burden of cerebrovascular conditions [7,8,9].

The U.S. is experiencing a decline in the incidence of stroke and other cerebrovascular conditions in Whites but not in Blacks [10]. Compared to White Americans, Black Americans are at higher risk of stroke [11] and associated mortality [12]. Stroke occurs at an earlier age among Blacks compared to Whites [11]. The relative excess stroke mortality among Blacks compared with Whites is most marked in the population aged less than 65 years [12]. Among men 45 to 54 years old, stroke death is 3.7 times more common in Blacks compared to Whites [12]. Higher mortality of stroke among Blacks is in part due to higher severity of stroke in Blacks than Whites [12,13]. Some of the epidemiological risk factors of the excess stroke mortality of Blacks are low socioeconomic status (e.g., education and income) [14], medical risk factors (e.g., hypertension) [15,16,17], and behavioral factors such as obesity [18].

Obesity is one of the potential mechanisms behind racial disparity in stroke mortality [12]. Obesity, which increases risk of stroke [19,20,21,22,23,24,25,26,27,28,29,30], is more common among Blacks than Whites [18]. Every 5 kg/m^2^ additional body mass index (BMI) is associated with 18% additional risk of stroke after adjustment for demographic and socioeconomic confounders [19]. 

With a higher prevalence among Blacks, obesity may be one of the factors explaining racial disparities in cerebrovascular mortality in the U.S. [19]. However, it is still unknown whether obesity differentially increases risk of cerebrovascular mortality in Blacks and Whites. Given the growing literature on Black–White differences in the effects of socioeconomic [10,12], psychological [19,31,32,33,34,35,36] and medical [37] risk factors on health outcomes, Blacks and Whites may also differ in the salience of obesity on future risk of cerebrovascular mortality. Social class and race are shown to alter the effects of obesity, hostility, and depression on mortality due to diabetes (DM), cardiovascular disease, and renal disease [31,36,37,38]. One reason behind the differential effect of baseline risk factors on long-term health outcomes is differential stability of risk factors over time [39]. For instance, Black smokers have a higher chance of quitting, due to lower severity of smoking, compared to Whites [39]. As a result, risk factors have differential effects on health outcomes across demographic and social groups [33]. In addition, obesity has different correlates in Whites and Blacks [40,41,42,43]. Finally, Blacks and Whites with obesity also have different levels of intention for weight loss [44,45]. 

### 1.2. Aim

To extend the existing knowledge on how risk factors operate across racial and ethnic groups, this study compared Blacks and Whites for the effect of baseline obesity on risk of cerebrovascular mortality over a 25-year period. 

## 2. Methods

### 2.1. Design and Setting

The Americans’ Changing Lives (ACL) study is a 25-year longitudinal study in the U.S. The ACL was conducted from 1986 to 2011. More information about design, methods, and sampling of the ACL has been published previously [46,47,48]. 

### 2.2. Study Population

ACL used a stratified multistage probability sampling to recruit adults 25 years old or above. All participants were living in the continental U.S. in 1986. The ACL included 3617 non-institutionalized people. The ACL study oversampled older adults (those age 60 and older) and Blacks. The ACL successfully recruited 68–70% of sampled individuals and households, respectively. Participants entered the ACL and the current analysis regardless of their risk factor profile including but not limited to obesity, substance use, smoking, and alcohol use.

### 2.3. Ethics

The ACL study protocol was approved by the University of Michigan institutional review board (# AG018418). Informed consent was received from all ACL participants.

### 2.4. Measures

Data were collected on race, demographic characteristics, socioeconomic factors, and chronic medical conditions (CMC) at the baseline face-to-face interview in 1986. Mortality due to cerebrovascular causes was collected from 1986 to 2011. Baseline obesity was the predictor of interest, and cerebrovascular mortality was the main outcome.

#### 2.4.1. Exposure

Obesity, defined as a BMI larger than 30 kg/m^2^, was the independent variable. In this study, BMI was measured based on self-reported height and weight. BMI based on self-reported data correlates strongly with measured BMI [49,50,51,52].

#### 2.4.2. Outcome

Mortality due to cerebrovascular diseases was the main outcome in this study. Information on mortality during the 25 years of follow up was obtained via death certificates, National Death Index (NDI), and the informants (spouse, partner, etc). In most cases, death certificates were enough to verify the time and cause of death. In a handful of cases where death certificates were not enough to verify death, other sources were reviewed for additional information. Occurrence of death was certain in all of the deceased cases. In a handful of cases, however, the date of death was determined by asking an informant or from the NDI report, but not the death certificates [53,54]. 

In this study, to classify various causes of death, we applied the ICD 9 and 10 codes [55,56]. Cause of death was considered as missing if the death certificate and NDI report could not be achieved. ICD 10 as well as ICD 10 systems were used to determine cause of mortality, depending on whether the time of death had been recorded. 

#### 2.4.3. Confounders

Baseline demographic characteristics, socioeconomic factors, health behaviors, and health status were covariates in this study. 

*Demographic Factors.* Demographic indicators included age and gender (men as the reference group).

*Socioeconomic Status (SES) Characteristics.* In this study, SES indicators included educational attainment and household income. Educational attainment was a dichotomous variable: 0–11 years of schooling = 0, and 12+ years of schooling = 1. Annual household income was also measured.

*Exercise (Physical Activity)*. To measure physical activity, ACL used a physical activity index, which evaluated how often respondents were engaged in the following three types of activities: (1) taking walks, (2) participating in exercise or active sports, and (3) working in the garden or yard. A four level Likert scale item response was used ranging from “often” to “never.” The physical activity score was calculated as the mean of the three items [57]. Higher scores indicated a higher level of physical activity [58].

*Smoking (i.e., tobacco use).* To measure smoking status, respondents were asked whether they smoke at the time of survey. A dummy variable was defined where non-smokers were the referent category [58].

*Depressive Symptomatology.* Symptoms of depression were evaluated using the Center for Epidemiological Studies-Depression scale (CES-D) 11-item version [59]. This scale evaluates how much the respondents had felt happy, depressed, lonely, sad, and had restless sleep and somatic symptoms. Abbreviated CES-D measures are both reliable and valid and have similar factor structure compared to the original CES-D scale [60,61,62]. Item response categories were 1 (“hardly ever”) to 3 (“most of the time”). All of the positive items were recoded. Mean CES-D scores were then calculated using all eight items. Thus, baseline depressive symptoms ranged from 1 to 3. Higher CES-D scores were indicative of a greater severity of depressive symptomatology.

*Hypertension (HTN)*: Hypertension and six other chronic medical conditions were measured at baseline using a self-reported measure [46,63,64,65]. Participants were asked whether a health care provider had ever told them that they had a certain chronic disease [53,64].

#### 2.4.4. Moderator

*Race.* Race was the focal moderator in this study. In this study, race was operationalized as a dichotomous variable, either non-Hispanic Whites or non-Hispanic Blacks [reference category]. Race and ethnicity were self-reported and measured at baseline in the year 1986 using several survey items. Hispanics were omitted from this analysis.

### 2.5. Statistical Analysis

Our univariate, bivariate, and multivariable data analyses were performed in Stata 13.0 (Stata Corporation, College Station, TX, USA). Due to the complex survey design (due to the multistage sampling that involved clustering and stratification) of the ACL, Taylor series linearization was used to estimate the standard errors using the sampling weights (due to stratification, clustering, and non-response). All proportions have applied weights. Mean and frequency tables were used to describe our sample. Chi square and independent sample t tests were used for bivariate analyses. A sub-population Cox regression analysis was applied, as our sample was limited to Whites and Blacks.

For the purpose of multivariable analysis, we applied Cox proportional hazards modeling to test if the association between obesity and cerebrovascular mortality is independent of confounders. A list of potential confounders of the association between obesity and cerebrovascular mortality were selected as a priori based on an extensive literature review that showed demographics, socioeconomic factors, health behaviors, and health status should be controlled. In all models, age, gender, education, income, exercise, smoking, hypertension, self-rated health, and depressive symptoms were controlled.

Cox proportional hazards models require an event (death due to cerebrovascular disease) and the time to event (time to death due to cerebrovascular disease). A cerebrovascular cause of death was considered 0 for the respondents who did not die or died due to any other cause of death. Time to the cerebrovascular death event or censoring was calculated as the number of months that had passed from baseline to death, loss to follow up, or the end of the longitudinal cohort study (year 2011).

*Model 1* was performed in the pooled sample and included the main effects. 

Log h(t) = f[h_0(t), alpha + beta_1_ race + beta_2_ age + beta_3_ gender + beta_4_ education + beta_5_ income + beta_6_ smoking + beta_7_ exercise + beta_8_ depression + beta_9_ HTN + beta_10_ obesity. 

*Model 2* was run in the pooled sample and included the race by obesity interaction as well. 

Log h(t) = f[h_0(t), alpha + beta_1_ race + beta_2_ age + beta_3_ gender + beta_4_ education + beta_5_ income + beta_6_ smoking + beta_7_ exercise + beta_8_ depression + beta_9_ HTN + beta_10_ obesity + beta_11_ obesity * race. 

*Model 3* and *Model 4* were performed in White and Black people, respectively. 

Log h(t) = f[h_0(t), alpha + beta_1_ age + beta_2_ gender + beta_3_ education + beta_4_ income + beta_5_ smoking + beta_6_ exercise + beta_7_ depression + beta_8_ HTN + beta_9_ obesity. 

We also ran similar models with overweight (BMI > 25) instead of obesity (BMI > 30) as the exposure. 

From all these models, hazard ratios (HR) [the values of Log(beta_n_)] and their associated 95% confidence intervals (CI) were reported from all of the above models. A *p* value less than 0.05 was regarded as statistically significant. The threshold of the *p* value was not adjusted as only four regression models were performed. Missing data were less than 5%.

## 3. Results

### 3.1. Descriptive Statistics

This study followed 3361 individuals for up to 25 years, from which 1156 were Blacks and 2205 were Whites. Table 1 summarizes the descriptive statistics for the pooled sample, and then separately for Whites and Blacks. Blacks were younger, had higher number of chronic medical conditions at baseline in comparison to Whites. Relative to White people, Black individuals had also lower educational attainment (*p* < 0.05 for all). Blacks also reported worse self-rated health (SRH) than Whites (Table 1).

The overall prevalence of DM was 5.73%, (95%CI = 4.80–6.82). DM was more common in Blacks (9.22%, 95%CI = 7.75–10.95) than Whites (5.25%, 95%CI = 4.24–6.50). This difference was significant at a p of 0.05. Similarly, overall, people had 12.53 years of schooling at baseline (95% CI = 12.34–12.73). A comparison of racial groups showed higher educational attainment in Whites (12.69, 95% CI= 12.48–12.90) than Blacks (11.37, 95% CI = 10.90–11.84). Thus, on average, Whites had more than 1.3 years higher years of schooling than Blacks, which was statistically significant at a p of 0.05.

### 3.2. Cerebrovascular Deaths

Of the 177 that died, 121 were White (68.36%) and 56 were Black (31.64%). Of the 177 that died, 33 were obese (18.64%) and 144 were not obese (81.36%) at baseline.

In bivariate association, race was not associated with death due to cerebrovascular (unadjusted HR for Blacks compared to Whites = 0.78, 95% CI = 0.55–1.11), suggesting that Whites and Blacks had similar risk of future cerebrovascular mortality over 25 years.

In bivariate association, baseline obesity was not associated with future risk of cerebrovascular mortality (Unadjusted HR for Blacks compared to Whites = 0.84, 95% CI = 0.45–1.56), suggesting that Whites and Blacks had a similar risk of future cerebrovascular mortality over 25 years.

### 3.3. Bivariate Correlations

Table 2 presents bivariate correlation between the study independent variables, moderator, confounders, and the outcome. Race (Black) was negatively associated with education and income but positively associated with depressive symptoms, hypertension, and obesity. Blacks more frequently smoked and less frequently exercised. Race was not associated with cerebrovascular death. Baseline obesity was associated with female gender and less education, income, smoking, and exercise. Obesity at baseline was associated with depressive symptoms and hypertension at baseline. Obesity at baseline was not associated with cerebrovascular death in the pooled sample (Table 2).

### 3.4. Models in the Pooled Sample

Table 3 summarized the results of *Model 1* and *Model 2*. According to *Model 1* in the pooled sample, baseline obesity did not predict cerebrovascular mortality (HR = 0.86, 0.49–1.51), independent of demographic, socioeconomic, health behaviors, and health factors at baseline. According to *Model 2***,** race interacted with baseline obesity on outcome (HR = 3.17, 1.09–9.21), suggesting a stronger association between baseline obesity and future risk for cerebrovascular deaths for Blacks, in comparison to Whites (Table 3).

### 3.5. Models in Whites and Blacks

Table 4 summarized the results of Model 3 and Model 4 in Whites and Blacks, respectively. As Model 3 shows, obesity did not predict the outcome in Whites (HR = 0.69, 0.31–1.53). Model 4 shows that obesity predicts risk of cerebrovascular mortality for Blacks (HR = 2.51, 1.43–4.39) (Table 4).

## 4. Discussion

According to our findings, race interacts with baseline obesity on long-term risk of cerebrovascular mortality. Compared to White individuals, Black people may be more vulnerable to the effect of baseline obesity on cerebrovascular mortality over a 25-year period. Given the representative nature of the ACL sample, the results are generalizable to the U.S. general population.

This study introduces obesity as a risk factor that disproportionately contributes to the risk of cerebrovascular conditions among Blacks [11,12,13]. Our finding emphasizes the role of obesity as an established risk factor for stroke and other cerebrovascular conditions [19,20,21,22,23,24,25,26,27,28,29,30]. This finding suggests that obesity may be one of the explanatory factors behind Black–White disparities in stroke mortality in the U.S. 

Our findings are in line with a growing literature on differential effects of risk factors on a wide range of health outcomes [31,32,33,34,35,36,37,66,67,68,69]. For social risk factors, however, the effects are systematically larger for Whites than Blacks [70]. In our study, however, obesity was a stronger risk factor for Blacks than Whites.

According to a joint statement by the American Stroke Association (ASA) and American Heart Association (AHA), Blacks and other minorities arrive later to an emergency department, wait longer in the emergency department, and have a lower chance of receiving thrombolysis for acute ischemic stroke. Differential treatment, defined as the presence of bias in the delivery of care is also important. Minorities experience longer stays in rehabilitation services and have poorer functional status compared to Whites. Minorities do not receive equal treatment for either primary or secondary prevention of stroke compared with Whites [70].

Disproportionate prevalence of obesity between Whites and Blacks may explain racial disparities in stroke mortality [12]. Obesity, which increases risk of stroke [19,20,21,22,23,24,25,26,27,28,29,30], is more common among Blacks [18]. Each 5 kg/m^2^ higher BMI is associated with 18% additional risk for stroke after adjustment for sociodemographic confounders. Additional control for the three metabolic risk factors (i.e., blood pressure, cholesterol, and glucose) reduces the additional risk of stroke to 4%, suggesting that more than three quarters of the additional risk of BMI for stroke is due to blood pressure, cholesterol, and glucose. From these metabolic risk factors, blood pressure seemed to be the most salient explanatory factor, as it accounted for 65% of the excess risk for stroke. Both overweight (BMI between 25 and 30 kg/m^2^) and obesity (BMI equal or larger than 30 kg/m^2^) are associated with an additional risk of stroke, compared to the individuals with normal weight (BMI between 20 and 25 kg/m^2^). The study showed that 98% and 69% of the excess risk of stroke due to overweight and obesity is mediated by glucose, blood pressure, and cholesterol level [19].

Our findings support the “differential vulnerability” theory [32,67,68,69,70,71,72,73], which conceptualizes race, ethnicity, gender, SES, and place as contextual effect modifiers that alter susceptibility to a wide range of psychosocial risk factors on health outcomes including but not limited to mortality. In this view, race, gender, class, place, and social, behavioral, psychological, and medical risk factors have multiplicative rather than additive effects. That is, the effect of each risk factor is specific to the sub-population.

The evidence is still mixed regarding factors that explain the higher burden of metabolic, cardiac, and cardiovascular outcomes. The differential effect of risk factors is not specific to cerebrovascular disease and extends to all-cause mortality [32,67,71], renal disease mortality [37,72] and cardiovascular conditions [36,38]. Thus, as a general rule, one size does not fit all, and groups differ in social, behavioral, psychological, and medical determinants of health and illness.

In the US, race is a proxy of social class and socioeconomic status. As a result, most racial disparities in health are a function of sociological rather than biological factors [74]. Studies have shown that the predictive power of risk factors on mortality depend on class [75] and race [32,34,66,67]. Therefore, our findings on the interaction between race and obesity on mortality may be due to socioeconomic factors or race itself. This differential effect may be due to a higher chronicity of obesity in Blacks [44,45,76,77].

Blacks and Whites may have different body images and may have different tolerances of obesity. This is evident by research showing that obese Blacks have lower depression and are less frequently interested in weight loss. Blacks and Whites may have different levels of error in reporting weight and height. Due to cultural issues, cognitive styles, socioeconomics, health literacy, numeracy skills, and validity of self-reported BMI may differ between Blacks and Whites. However, a lower validity of BMI would not strengthen the predictive role of obesity on mortality for Blacks. Based on more tolerance, the author expected a lower predictive role of BMI for Blacks. However, our finding is not in agreement with the general pattern reported before [46,78,79,80] and the Black–White health paradox [81,82].

Mehta et al. used the cross-sectional NHANES data to examine the interactions between five mortality risk factors, namely race, sex, educational attainment, smoking, and obesity [52]. The study showed that several of these behavioral risk factors, including obesity, have differential effects across demographic and socioeconomic groups [52]. Assari has also summarized the results of more than 40 papers that show differential effects between Whites and Blacks [70]. Williams and Kessler have emphasized the need for systematic investigation of interactions between race and risk factors on health [83,84]. Kaufman, however, has discussed the difficulty of such an approach due to a wide range of potential methodological biases, such as residual confounding and the association of race with the distribution of several risk factors [85].

Differential vulnerability is a neglected aspect of health disparities research. Differential vulnerabilities may contribute to higher burden of cerebrovascular disease in Blacks. Most of the previous focus has been on higher exposure to risk factors such as hypertension, obesity, and other factors such as SES, health care use, and differential treatment [11,12]. Thus, there is still a need for more research on the role of the differential vulnerability of Blacks and Whites.

Although obesity was associated with a larger effect on the long-term risk of cerebrovascular mortality for Blacks than Whites, the literature shows that Whites may be more vulnerable to the effects of education on both obesity [86] and hospitalization [87]. In a study, educational attainment better predicted the risk of all-cause mortality for Whites than Blacks [67]. This paradoxical pattern requires additional investigation. 

We found a higher prevalence of obesity in Blacks than Whites. The environment is more obesogenic for Blacks than Whites, and we discussed this issue. A larger proportion of Blacks live in urban areas that are characterized by food deserts, fast foods, poor park and green spaces. There are a few citations regarding the diet-induced obesity difference between Blacks and Whites [88]. In addition, southern Black culture is linked to obesogenic food [89].

### 4.1. Limitations

The first and foremost limitation of our study was lack of data on any biological marker, such as cholesterol, glucose, arterial thickening, etc. The study did not collect data on the number or nature of cerebrovascular events, as well as their treatments. It is unclear if Blacks are more likely to have cerebrovascular events or they are more likely to die because of cerebrovascular events due to bias of treatment effect. The study did not collect data on a wide range of medical conditions such as history of stroke. The study also measured hypertension based on self-reported data and may be subjected to recall bias. Data were also old, thus there is a need to replicate these finding using more recent data. In addition, Hispanics were removed from the analysis, as our line of research has compared non-Hispanic White and non-Hispanic Blacks for the effect of risk and protective factors. This needs to be clearly stated and justified. Future research should also validate BMI based on self-reported height and weight across racial groups. Finally, BMI, HTN, and other study variables were subject to change over time, and its change was not modeled in this study. Despite these limitations, our results are an extension of the existing literature on racial health disparities in stroke and other cerebrovascular conditions. Major strengths of this study included a (1) nationally representative sample, (2) large sample size, and (3) long-term follow up.

### 4.2. Implications

Our findings may have some clinical and public health implications for reducing racial disparities in cerebrovascular mortality in the U.S. Our findings suggest that universal intervention for prevention of obesity may have differential effects in reducing cerebrovascular mortality in Whites and Blacks.

These results are important given the increasing epidemics of obesity in the U.S. [60,76]. Stroke, cerebrovascular, and cardiovascular disease is the largest contributor to the Black–White health disparities in life expectancy. A higher burden of stroke in Blacks compared to Whites is persistent [10,90]. A population-based study in the U.S. showed that the incidence of ischemic stroke declined from 1993 to 2010 in Whites [91], however, the incidence did not decline among Blacks, which was suggestive of widened racial disparities in stroke incidence [91]. Other studies have reported similar findings [10].

### 4.3. Future Research

Our results suggest directions for future research. Future studies should examine whether obese Whites and Blacks die from different causes or not. The role of competing risks such as cardiovascular conditions are still unknown. There is a need for replication of these findings in other large data sets. Finally, it is still unclear which behaviors or factors explain the disproportionate risk of mortality of Whites and Blacks from certain causes including but not limited to cerebrovascular events.

Differential vulnerability may be a neglected contributing factor to the higher burden of cerebrovascular disease among Blacks and other minority groups [70]. A single risk factor may have a weaker or stronger effect on health conditions across racial and ethnic groups. The links between race, ethnicity, social class, risk factors, and health outcomes such as mortality are not additive but multiplicative and non-linear. Race not only changes the exposure but also the vulnerability and susceptibility to the risk and protective factors. A better understanding of heterogeneities in the effects of risk factors needs further research.

## 5. Conclusions

Race may alter the long-term effect of baseline obesity on deaths due to cerebrovascular disease. In the U.S., obesity may be differently salient for cerebrovascular disease mortality for Blacks than Whites.

## Figures and Tables

**Table 1:** Descriptive statistics in the pooled sample and also based on race (*n* = 3361).

Characteristics	Pooled Sample (*n* =3361)			White Individuals (*n* = 2205)		Black Individuals (*n* = 1156)
	M	95% CI	M	95% CI	M	95% CI
**Demographics**						
Age (Years)	47.79	46.72–48.86	47.98	46.77–49.19	46.37	44.93–47.81
	%	95 % CI	%	95 % CI	%	95 % CI
Gender						
Men	47.26	44.86–49.68	47.82	45.12–50.52	43.18	38.79–47.69
Women	52.74	50.32–55.14	52.18	49.48–54.88	56.82	52.31–61.21
**Socioeconomics**						
Education *						
11 Years or Less	23.93	21.37–26.70	21.71	18.87–24.85	40.25	34.55–46.24
>12 Years	76.07	73.30–78.63	78.29	75.15–81.13	59.75	53.76–65.45
	M	95% CI	M	95% CI	M	95% CI
Education *	12.53	12.34–12.73	12.69	12.48–12.90	11.37	10.90–11.84
Income ($1000) *	5.41	5.22–5.60	5.57	5.36–5.77	4.25	3.88–4.62
**Health**						
Self-Rated Health *	2.30	2.25–2.35	2.28	2.23–2.33	2.43	2.32–2.54
Chronic Medical Conditions*	0.80	0.74–0.85	0.78	0.71–0.84	0.92	0.82–1.02
	%	95 % CI	%	95 % CI	%	95 % CI
**Health**						
Self-Rated Health *						
Good or Excellent	85.06	83.33–86.64	85.97	84.15–87.60	78.38	74.68–81.68
Poor or Fair	14.94	13.36–16.67	14.03	12.40–15.85	21.62	18.32–25.32
Smoking *	30.45	27.81–33.23	29.70	26.85–32.72	35.98	30.81–41.49
Obesity *						
No	85.54	83.76–8714	86.48	84.47–88.27	78.56	74.50–82.13
Yes	14.46	12.86–1624	13.52	11.73–15.53	21.44	17.87–25.50

M: Mean, CI: Confidence Interval, * *p* < 0.05.

**Table 2:** Correlation matrix of the study variables (*n* = 3361).

Characteristics	1	2	3	4	5	6	7	8	9	10	11
1 Race	1.00 *										
2 Age	−0.06 *	1.00									
3 Gender	0.05 *	0.11 *	1.00								
4 Education	−0.26 *	−0.39 *	−0.06 *	1.00							
5 Income	−0.29 *	−0.30 *	−0.20 *	0.52 *	1.00						
6 Smoking	0.06 *	−0.21 *	−0.07 *	0.00	−0.02	1.00					
7 Exercise	−0.16 *	−0.24 *	−0.16 *	0.28 *	0.27 *	−0.05 *	1.00				
8 Depressive Symptoms	0.17 *	−0.07 *	0.11 *	−0.19 *	−0.24 *	0.09 *	−0.21 *	1.00			
9 HTN	0.16 *	0.36 *	0.11 *	−0.28 *	−0.25 *	−0.11 *	−0.20 *	0.09 *	1.00		
10 Obesity	0.14 *	0.02	0.08 *	−0.13 *	−0.10 *	−0.05 *	−0.12 *	0.05 *	0.21 *	1.00	
11 Death to cerebrovascular disease	−0.01	0.21 *	0.04 *	−0.07 *	−0.09 *	−0.06 *	−0.07 *	0.02	0.07 *	0.01	1.00

* *p* < 0.05.

**Table 3:** Pooled sample Cox proportional hazards models with time of death due to cerebrovascular disease as the outcome (*n* = 3361).

Characteristics	HR (SE)	95%	HR (SE)	95%
	Model 1 Main Effect Model		Model 2 Model with Interaction	
**Demographics**				
Age (Years)	1.14(0.01) ***	1.12–1.17	1.14(0.01) ***	1.12–1.17
Gender (Women)	0.57(0.13) *	0.36–0.91	0.57(0.13) *	0.36–0.90
Race (Blacks)	0.78(0.17)	0.50–1.22	0.57(0.16) *	0.33–1.00
**Socioeconomics**				
Education (Low)	0.99(0.04)	0.91–1.07	0.98(0.04)	0.91–1.07
Income (Low)	0.92(0.05)	0.82–1.04	0.92(0.05)	0.82–1.04
**Health Behaviors**				
Smoking	1.53(0.41)	0.89–2.62	1.51(0.40)	0.88–2.59
Exercise	1.02(0.10)	0.84–1.23	1.01(0.10)	0.84–1.23
**Health**				
Depressive Symptoms (>M+SD)	1.22(0.10) *	1.04–1.44	1.23(0.10) *	1.04–1.45
Hypertension	1.41(0.30)	0.91–2.17	1.41(0.31)	0.91–2.19
**Obesity**				
Obese	0.86(0.24)	0.49–1.51	0.69(0.27)	0.31–1.53
Obese × Race (Blacks)	–	–	3.17(1.68) *	1.09–9.21

* *p* < 0.05, ** *p* < 0.01, *** *p* < 0.001.

**Table 4:** Stratified Cox proportional hazards models with time to death due to cerebrovascular disease as the outcome.

Characteristics	Model 3 Whites (*n* = 2205)		Model 4 Blacks (*n* = 1156)	
	HR (SE)	95%CI	HR (SE)	95%CI
**Demographics**				
Age (Years)	1.15(0.02) ***	1.12–1.19	1.10(0.02) ***	1.07–1.13
Gender (Women)	0.59(0.14) *	0.36–0.95	0.35(0.11) **	0.19–0.67
**Socioeconomics**				
Education (Low)	0.96(0.05)	0.88–1.06	1.09(0.04) *	1.01–1.17
Income (Low)	0.94(0.06)	0.82–1.07	0.78(0.06) **	0.67–0.91
**Health Behaviors**				
Smoking	1.64(0.48) #	0.92–2.94	0.73(0.32)	0.30–1.76
Exercise	1.06(0.10)	0.87–1.29	0.66(0.13) *	0.44–0.98
**Health**				
Depressive Symptoms (>M + SD)	1.24(0.11) *	1.03–1.49	1.26(0.20)	0.91–1.74
Hypertension	1.52(0.34) #	0.96–2.40	0.61(0.20)	0.31–1.20
**Obesity**				
Obese	0.69(0.27)	0.31–1.53	2.51(0.70) **	1.43–4.39

# *p* < 0.1, * *p* < 0.05, ** *p* < 0.01, *** *p* < 0.001.
